# Role of *Candida albicans* on enamel demineralization and on acidogenic potential of *Streptococcus mutans in vitro* biofilms

**DOI:** 10.1590/1678-7757-2018-0593

**Published:** 2019-08-30

**Authors:** Gustavo Eidt, Caroline Gomes de Andrade, Thais de Cássia Negrini, Rodrigo Alex Arthur

**Affiliations:** 1 Universidade Federal do Rio Grande do Sul Universidade Federal do Rio Grande do Sul Faculdade de Odontologia Departamento de Odontologia Preventiva e Social Porto Alegre Rio Grande do Sul Brasil Universidade Federal do Rio Grande do Sul, Faculdade de Odontologia, Departamento de Odontologia Preventiva e Social, Porto Alegre, Rio Grande do Sul, Brasil

**Keywords:** *Candida albicans*, *Streptococcus mutans*, Biofilms, Dental caries, Dental enamel

## Abstract

There is growing evidence that *C. albicans* is associated with dental caries, but its role on caries development needs to be better clarified.

**Objective:**

To evaluate at the hard tissue level the effect of *C. albicans* on the cariogenic potential of *S. mutans* biofilms focusing on the mineral profile of induced carious lesions. This study also aimed to evaluate the effect of *C. albicans* on the acidogenic potential of *S. mutans* biofilms.

**Methodology:**

Dual-species (CA+SM) and single-species biofilms (CA or SM) were grown on the surface of enamel slabs in the presence of glucose/sucrose supplemented culture medium for 24, 48 and 72 hours. Demineralization was evaluated through percentage of surface microhardness change (%SMC) and transversal microradiography analysis (ILM and LD) and pH of the spent medium was recorded daily. Data were analyzed by two-way ANOVA followed by Bonferroni correction.

**Results:**

%SMC was statistically different among the biofilms at each time point being the highest for SM biofilms and the lowest for CA biofilms which also differed from CA+SM biofilms [SM (24 h: 47.0±7.3; 48 h: 66.3±8.3; 72 h: 75.4±3.9); CA (24 h: 7.3±3.3; 48 h: 7.1±6.4; 72 h: 6.6±3.6); CA+SM (24 h: 35.9±7.39.1; 48 h: 47.2±9.5; 72 h: 47.6±9.5)]. pH of spent medium was statistically lower for SM biofilms compared to the other biofilms at each time point and remained constant over time while pH values increased from 24 to 72 h for both CA and CA+SM biofilms [SM (24 h: 4.4±0.1; 48 h: 4.4±0.1; 72 h: 4.5±0.1); CA (24 h: 6.9±0.3; 48 h: 7.2±0.2; 72 h: 7.5±0.2); CA+MS (24 h: 4.7±0.2; 48 h: 5.1±0.1; 72 h: 6.1±0.6)]. IML and LD for SM biofilms increased over time while no difference was observed from 24 to 72 h for the other biofilms.

**Conclusions:**

The present data suggest that *C. albicans* has low enamel demineralization potential and the presence of *C. albicans* can reduce both the cariogenic and acidogenic potentials of *S. mutans* biofilms.

## Introduction

Acid production, through fermentation of carbohydrates by a selective group of microorganisms, results in pH reduction within biofilms. This acidification is directly related to the dissolution of tooth tissues that might occur if the balance between demineralization and remineralization process is disturbed over an extended period.^1^ So, in dental caries, frequent sugar intake results in a dysbiotic change in dental plaque where the acid-tolerant microorganisms prevail over the less acid-tolerant ones in response to biofilm acidification.^2^

*Candida albicans* has a high acid tolerance and is capable of producing acids even under low pH conditions. These characteristics may favor the fungus in the microbial shifts associated with dental caries.^3^^,^^4^ In fact, the frequency of isolation of *C. albicans* is significantly higher in biofilms associated with carious teeth.^5^^–^^9^

Furthermore, one of the microorganisms most frequently associated with dental caries is the bacterial species *S. mutans.* Despite being not alone in this task, *S. mutans* has a high acid production ability. In addition, it has the unique ability of converting sucrose into extracellular insoluble glucans (EPS) that constitute the main component of the biofilm matrix. Insoluble glucans alter the diffusion properties of biofilm matrix by acting as a barrier to the buffering effects of saliva. Additionally, they can promote the establishment of acidic microenvironments and thereby the formation of acidic niches that exhibit an increased demineralization potential.^10^^–^^12^

Literature has extensively provided evidences that biofilms formed by *C. albicans* and *S. mutans* present increased cariogenic potential compared to single-species biofilms. Previous studies have focused either on biological mechanisms related to such increased cariogenic potential and on changes induced by this microbial association on the three-dimensional structure of biofilms.^13^^–^^18^ Rodent models have also been used to show that carious lesions produced by the association between *C. albicans* and *S.* *mutans* are more severe.^13^ Therefore, a positive association between the presence of *C. albicans* and the development of cariogenic biofilms may exist, especially in severe conditions such as early childhood caries.^19^^–^^21^

However, the role played by this microbial association on dental caries onset is still controversial since other studies have indicated opposite findings.^22^^,^^23^ Yet, the association between surrogate outcomes, such as the carriage of streptococci and *C. albicans* on dental plaque and saliva, and caries onset^21^ is questioned.^24^ Additionally, to the best of our knowledge, no other study has objectively assessed the profile of enamel mineral loss in the presence of that microbial association.

Thus, the aim of this study was to evaluate at the hard tissue level the effect of *Candida albicans* on the cariogenic potential of *Streptococcus mutans* biofilms focusing on the mineral profile of induced carious lesions. Additionally, this study aimed to evaluate the effect of *C. albicans* on the acidogenic potential of *Streptococcus mutans* biofilms. To this end, an *in vitro* biofilm model was used to induce enamel carious lesions that were assessed via surface hardness and whose mineral profile was evaluated by transversal microradiography. The null hypothesis was that *C. albicans* do not affect the cariogenic and acidogenic potentials of *S. mutans* biofilms.

## Methodology

### Experimental design

Dual-species (MIX; *C. albicans + S. mutans; n*=24) and single-species (*C. albicans; n*=24 or *S. mutans; n*=24) biofilms were grown on the surface of sound bovine enamel slabs in the presence of culture medium supplemented with 6 mM glucose and 3 mM sucrose for 24, 48 and 72 hours (n=8 for each biofilm experimental period). The medium was refreshed daily and the pH of the spent medium was recorded. Biofilms were harvested and viable cell counts were performed at the end of each experimental period. Enamel demineralization and mineral profile were evaluated through the determination of percentage of surface hardness change, via hardness analysis, and integrated mineral loss and lesion depth, via transversal microradiography. The experiments were done in triplicate.

### Enamel slabs preparation

Enamel slabs were cut from sound bovine anterior teeth, which were previously disinfected in 10% formaldehyde solution,^25^ using a drill bench (Schulz S.A., Joinville, SC, Brazil) coupled with a ¼ inch diamond grit hole saw (DeWALT, Baltimore, MD, USA). The resulting 6.4 mm diameter enamel slabs were then flattened and polished with a grinding and polishing machine (Arotec S/A, Cotia, SP, Brazil). A 1 mm nail varnish strip was made on the side of each slab to create an unexposed (control) area. Slabs with cracks, scratches, exposed dentin or with mean enamel baseline hardness out of the range from 320 to 360 Vickers microhardness (VMH) were discarded. Baseline surface hardness determination is described below. Slabs were then randomized and balanced into experimental groups by mean VMH values. Finally, the slabs were mounted on individual nylon cylinders attached to the lid of 24-well plates^26^ and they were subjected to hydrogen peroxide gas sterilization.^27^

### Inoculum preparation

*Candida albicans* (ATCC 90028) and *Streptococcus mutans* (UA 159) were cultivated from frozen stocks on Brain Heart Infusion Agar (BHI; Kasvi, São José dos Pinhais, PR, Brazil). Colony-forming units (CFU) were then inoculated into tubes containing Tryptic Soy Broth (TSB; HiMedia, Mumbai, MH, India baseline pH from 7.1 to 7.3; 14 mM glucose basal concentration) supplemented with 12.5 mM sucrose. After 24 h of incubation, aliquots of each suspension were transferred into fresh medium and were further incubated for 24 h at 37°C. Optical density was adjusted independently for each microorganism to 0.5±0.1 at 550 nm with a spectrophotometer (Milton Roy Co., Ivyland, PA, USA), corresponding to approximately 10^7^ CFU/mL of *C. albicans* and 10^8^ CFU/mL of *S. mutans*. Counts of viable cells were confirmed at each experiment by plating the inoculum on BHI agar plates and incubating them under the conditions described above. Inoculum presented a mixture of hyphae and yeasts forms (qualitatively determined by microscopic analysis). Dual-species consortium was obtained by combining equal amounts of the density-adjusted cultures^26^.

### *In vitro* biofilm growth

The modified lids with the slabs were put onto 24-well plates with 0.4 mL inoculum and 1 mL TSB (pH 7; supplemented with 6 mM glucose and 3 mM sucrose; TSBGS; 20 mM glucose final concentration) on each well and kept at 37°C. After 8 h of incubation, the slabs were transferred to new plates containing 1.4 mL of TSBGS medium and incubated at 37°C overnight. Biofilms were then transferred every 24 hours to new 24-well plates containing fresh TSBGS medium and kept at 37°C^26^. The pH values of the spent medium (at 8, 24, 48 and 72 h) were individually measured for each well using a pH meter (Digimed, São Paulo, SP, Brazil) previously calibrated with pH 7.0 and pH 4.0 standards.

### Biofilm harvesting and viable cell counts

At the end of each experimental period (after 24, 48 and 72 h of biofilm growth), enamel slabs (*n*=8) were aseptically removed from the lids and individually transferred to tubes containing 1 mL of sterile 0.9% NaCl and 4 sterile glass beads (4-5 mm diameter). Tubes were vortexed for 30 s to disperse the biofilms. Aliquots of the microbial suspensions were serially diluted and plated on BHI agar. Plates were incubated at 37°C under microaerophilic conditions for 24 h. CFU were then counted under a stereomicroscope and the results expressed as CFU/mL. Enamel slabs were gently cleaned and stored in pre-codified vials at 4°C under humid environment until further assessment of caries lesion development.

### Surface microhardness

Baseline enamel surface hardness was determined by a microhardness tester (ISH-TDV2000; Insize Co. Ltd, Suzhou, JS, China) by making five Vickers indentations, spaced 100 µm from each other, with a load of 200 gf for 10 seconds. After each experimental period of biofilm growth, five indentations were placed 100 µm at the right side of the baseline indentations following the same parameters described above. The mean Vickers microhardness (VMH) values of baseline (B) and post-biofilm (P) VMH were averaged and the percentage of surface microhardness change (% SMC) was calculated as^26^ % SMC=[(P−B)÷B]×100.

### Transversal microradiography (TMR)

After post-biofilm hardness analysis, half of the specimens were sectioned through the center with a low-speed diamond saw (Isomer; Buehler Ltd., Lake Bluff, IL, USA), perpendicularly to the surface and transversally to the varnish strip, in order to obtain slices approximately 150 µm thick representing both exposed and unexposed (sound) enamel surfaces. Slices were then hand polished plane-parallel from both cut sides with 600 and 1200 grit sandpapers to a thickness of approximately 100 µm. Specimens were mounted in a custom-made sample-holder with an aluminium calibration step wedge with 14 steps. Microradiographs were taken with an X-ray generator (Softex Co. Ltd., Ebina, Japan) on a high precision glass plate (Konica Minolta Inc., Tokyo, Japan) at a distance of 42 cm using 20 kV and 20 mA for 13 minutes. Plates were developed for 5 min, rinsed with deionized water and fixed for 8 min in a dark room at 20°C. All plates were then washed in running water for 10 min and air-dried. Microradiographs were examined using a microscope (Carl Zeiss Microscopy GmbH, Jena, TH, Germany) in conjunction with a camera (Canon, Tokyo, Japan) and a computer running data-acquisition and calculation software programs (Inspektor Research Inc., Amsterdam, NH, Netherlands). The lesion depth (LD) was calculated using a threshold of 95% of the mineral content of sound enamel. Integrated mineral loss (IML; vol% mineral x µm) was also calculated.^28^ All analyses were performed by a blinded examiner.

### Statistical analysis

The mean and standard deviation (pH of spent medium, % SMC, LD and IML) were calculated for each tested condition. The assumptions of homogeneity of variances and normality of the distribution were checked. Counts of viable cells were log transformed and were descriptively reported as median and quartiles. Two-way analysis of variance (ANOVA) was used to determine the effects and interactions of the tested conditions (biofilm microbial composition and biofilm age) on response variables. Bonferroni adjusted comparisons were used when two-way ANOVA indicated statistically significant effects. Correlation between pH and counts of fungal viable cells on dual-species biofilms was tested using Pearson's coefficient, while correlation between %SMC and transversal microradiography outcomes was tested with Spearman's rank coefficient. All analyses were performed on SPSS Statistics for Windows, version 22.0 (IBM Corp. in Armonk, NY, USA) with a significance level set as 1%.

## Results

Regarding counts of viable cells, only changes lower than 1 log_10_ (10-fold) were found on median of CFU among the different tested conditions for both fungal and bacterial cells. Overall, CFU remained constant among different periods of biofilm growth irrespective to the biofilm composition (Table 1). For all the other outcomes (pH, %SMC, IML and LD) statistical analysis indicated an interaction effect between microbial composition and biofilm age (p<0.01).

**Table 1:** Viable cell counts [Log CFU/mL; median (25^th^/75^th^ quartiles)] according to biofilm age and biofilm microbial composition

Biofilm microbial composition					
Variable	Age	*C. albicans*		MIX	
		Median (25^th^-75^th^)	n	Median (25^th^-75^th^)	n
Fungi	24 h	6.4 (6.3 - 6.7)	24	6.3 (6.0 - 6.4)	24
	48 h	6.6 (6.3 - 7.2)	24	6.6 (6.4 - 6.7)	24
	72 h	7.1 (6.5 - 7.5)	24	6.7 (6.6 - 7.1)	24
Bacteria		***S. mutans***		**MIX**	
		**Median (25^th^-75^th^)**	**n**	**Median (25^th^-75^th^)**	**n**
	24 h	8.0 (7.9 - 8.1)	24	7.9 (7.7 - 8.0)	24
	48 h	8.0 (7.4 - 8.1)	24	7.7 (7.3 - 8.0)	24
	72 h	8.1 (7.7 - 8.2)	24	7.8 (7.2 - 8.2)	24

The mean of pH values after 8 hours of biofilm growth was 6.7±0.2, 4.8±0.1 and 4.4±0.1 for *C. albicans*, dual-species and *S. mutans* biofilms respectively. Spent medium pH related to *S. mutans* biofilms remained between 4.4 – 4.5 under all the tested experimental conditions being significantly lower than for other biofilms at each time point. The pH of dual-species biofilms increased over time being below the critical pH for enamel demineralization for up to 48 h of biofilm growth. Under all the tested conditions, pH of the spent medium of *C. albicans* biofilms slightly increased over time but remained higher than the critical pH needed to induce enamel carious lesions (Table 2). A moderate correlation between fungi CFU and spent medium pH (r=0.559; p<0.01) was found in dual-species biofilms.

**Table 2:** Spent medium pH, percentage of Surface Microhardness Change (%SMC), Integrated Mineral Loss (IML; Vol% x μm) and Lesion Depth (LD; μm) according to biofilm age and biofilm microbial composition

Biofilm microbial composition							
Variable	Age	*C. albicans*		MIX		*S. mutans*	
		Mean ± SD	n	Mean ± SD	n	Mean ± SD	n
pH	24 h	6.9 ± 0.3^Aa^	24	4.7 ± 0.2^Ab^	24	4.4 ± 0.1^Ac^	24
	48 h	7.2 ± 0.2^Ba^	24	5.1 ± 0.2^Bb^	24	4.4 ± 0.1^Ac^	24
	72 h	7.5 ± 0.2^Ca^	24	6.1 ± 0.6^Cb^	24	4.5 ± 0.1^Ac^	24
%SMC	24 h	7.3 ± 3.3^Aa^	24	35.9 ± 9.1^Ab^	24	47.0 ± 7.3^Ac^	24
	48 h	7.1 ± 6.4^Aa^	24	47.2 ± 9.5^Bb^	24	66.3 ± 8.3^Bc^	24
	72 h	6.6 ± 3.6^Aa^	24	47.6 ± 9.5^Bb^	24	75.4 ± 3.9^Cc^	24
IML	24 h	211.4 ± 60.9^Aa^	7	310.0 ± 140.8^Aa^	7	391.7 ± 146.5^Aa^	6
	48 h	276.7 ± 155.1^Aa^	9	667.5 ± 182.7^Aab^	8	1172.9 ± 478.3^Bb^	7
	72 h	238.6 ± 130.6^Aa^	7	596.3 ± 199.0^Aa^	8	2025.6 ± 862.9^Cb^	9
LD	24 h	11.4 ± 6.0^Aa^	7	15.1 ± 5.7^Aa^	7	19.6 ± 7.2^Aa^	6
	48 h	18.9 ± 16.8^Aa^	9	31.9 ± 9.5^Aab^	8	67.9 ± 60.2^ABb^	7
	72 h	13.3 ± 7.7^Aa^	7	32.0 ± 11.8^Aa^	8	103.0 ± 56.7^Bb^	9

Different uppercase letters show a significant difference between biofilm age and different lowercase letters show a significant difference between biofilm microbial compositions by two-way ANOVA followed by Bonferroni test (p<0.01)

In relation to carious lesion development, higher %SMC was found in the presence of *S. mutans* single-species biofilms than for other biofilms at each time point. In addition, *S. mutans* related demineralization increased with time, being statistically different among the different time points. On the other hand, carious lesion development in the presence of *C. albicans* single-species biofilms was statistically lower than in the presence of other biofilms and it remained constant over time. Additionally, the % SMC in the presence of dual-species biofilms at 48 h of biofilm formation was statistically higher than at 24 h, but it was not statistically different compared to enamel demineralization after 72 h of biofilm growth (Table 2).

At 24 h of biofilm growth, no statistical difference was found on IML and LD among the different biofilms, but enamel slabs exposed to *S. mutans* single-species biofilms presented IML and LD higher than slabs exposed to *C. albicans* single-species biofilms at 48 and 72 h and higher than dual-species biofilms at 72 h of biofilm growth. Furthermore, IML of enamel slabs exposed to *S. mutans* biofilms increased over time, being statistically different at each time point, whereas LD at 72 h was statistically higher than that found at 24 h of biofilm growth. Moreover, IML of enamel slabs exposed to *C. albicans* and to dual-species biofilms remained constant over time. This same behaviour was observed in relation to LD (Table 2).

**Figure 1:**
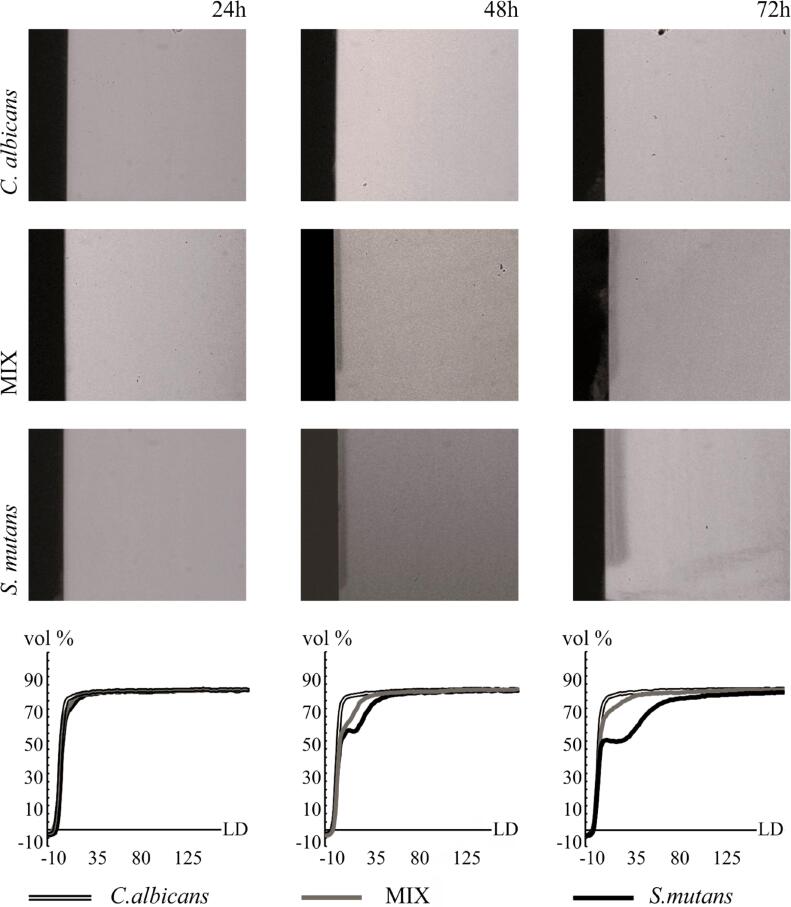
Representative microradiographs and mineral profile after 24, 48 and 72 hours of biofilm growth. Different coloured lines represent mean values of the percentage of mineral volume (vol %) according to lesion depth (LD) for each biofilm microbial composition

Figure 1 shows representative transversal microradiographs of enamel slabs according to the experimental conditions where it can be easily seen that all carious lesions were sub-superficial presenting a well-mineralized surface layer. Lesions formed in the presence of *S. mutans* biofilms were deeper and had lower mineral content compared to the other biofilms. Overall, a positive correlation was found between %SMC and IML (ρ=0.808; p<0.01) as well as between %SMC and LD (ρ=0.760; p<0.01).

## Discussion

As the presence of *C. albicans* has been increasingly related to dental caries,^21^ the present study used an *in vitro* biofilm model for the assessment of enamel carious lesion development at the hard tissue level by using surface hardness and transversal microradiography aiming to investigate the effect of *C. albicans* on the cariogenic potential of *S. mutans* biofilms as well as on their acidogenic potential.

It is well known that *S. mutans*, in addition to its acid-tolerance and acidogenic potential, can produce exoenzymes, known as glucosyltransferase B (GtfB), that synthesize EPS from sucrose, an essential factor associated to the cariogenic potential of biofilms.^10^,^12^,^29^ So, as expected, enamel slabs exposed to *S. mutans* single-species biofilms presented an increased mineral loss over time, taking both superficial microhardness and transversal microradiography outcomes into consideration (Table 2, Figure 1). On the other hand, less acidification was found in the presence of *C. albicans* single-species biofilms which lead to very little enamel demineralization (Table 2, Figure 1).

This low acidogenic potential found in *C. albicans* single-species biofilms could be related to the fact that this fungus may present a more complex acid production profile than lactic acid-producing bacteria.^3^ Pyruvate and acetate may be produced by this fungus in the presence of glucose alone, but almost no spent medium acidification has been found for *C. albicans* single-species biofilms grown in the presence of glucose and sucrose.^23^ Chemical characterization of *C. albicans* single-species related spent medium suggests minimum carbohydrate utilization and a poor ability of the fungus in metabolizing sucrose when growing alone.^17^ Additionally, it has been also suggested that in the presence of culture medium supplemented with sucrose, *C. albicans* may produce ethanol that does not influence the pH of the medium.^23^ Important to mention that the less acidified environment found in the presence of *C. albicans* could not be attributed to any colonization impairment or lack of fungi viability, since, although slightly changes had been found over time, CFU counts remained constant and similar to those of *S. mutans* throughout the experiments (Table 1).

It is important to acknowledge though that the low cariogenicity of *C. albicans* single-species biofilms found in this study might be strain-related and might also be a result of different biofilm growing conditions and/or experimental conditions compared to other studies since there are evidences showing that biofilms of *C. albicans* strains isolated from HIV+ children can cause enamel demineralization after 5 days of biofilm growth^30^^,^^31^ and that carious lesions are developed in rats infected with *C. albicans*.^13^^,^^22^ Nonetheless, it seems based on the present data that the isolated cariogenic potential of *C. albicans* in relation to enamel is not high.

Carbon source has a direct influence on biofilm morphogenesis.^32^^–^^34^ When *C. albicans* and *S. mutans* are growing together in mixed biofilms, they may compete for fermentable sugars. Under carbohydrate privation, *C. albicans* can use a variety of other carbon sources, such as amino acids, fatty acids and carboxylic acids.^35^ Breakdown of exogenous amino acids can lead to extrusion of ammonia, a highly basic compound. Consequently, in a glucose-limited milieu, *C. albicans* can actively modulate extracellular pH by alkalinisation of acidic environments.^36^

Additionally, *C. albicans* consumption of organic acids as carbon sources, such as pyruvate and lactate, is also responsible for the neutralization of acidic environments. It is likely that both mechanisms are acting together leading to the reduced acidification of dual-species biofilms over time. This way, in the presence of dual-species biofilms enamel slabs showed an initial caries lesion development, but no additional mineral loss could be observed after 48 hours (Figure 1). By providing data related to surface hardness and mineral profile of demineralized enamel specimens, our results corroborate with the results of Willems, et al.^23^ (2016) reinforcing that *C. albicans* reduce the cariogenic potential of *S. mutans* biofilms. It is important to highlight that Willems's study and ours used similar biofilm growth conditions (up to 72 hours in a medium with high protein and low carbohydrate contents). Thus, this reduced cariogenic potential may only occur in environments with high nutritional content but low carbohydrate concentrations.

One may argue that the reduced cariogenicity found on dual-species biofilms was a result of the less carbohydrate availability for biofilm growth, since the same sugar concentration was used for both single- and dual-species biofilms. It is important to emphasize though that the use of higher sugar concentration would lead to enamel surface softening compromising the assessment of enamel demineralization by means of surface hardness. Even having plenty of available sugars, *C. albicans* as single-species biofilm was not able to induce considerable enamel demineralization suggesting that the available sugar concentration was not the reason related to the reduced cariogenicity of dual-species biofilms. Moreover, it could also be argued that the increase on pH of spent medium of dual-species biofilms would be the result of a reduction on viable cells counts over time. It is important to emphasize that changes on counts of viable cells were too small (less than 1log_10_-fold) and do not present any relevance for caries development.

However, the data of the present study differ from previous studies. A more aggressive onset of carious lesions was found in the presence of dual-species biofilms.^13^ It has been shown that GtfB secreted by *S. mutans* adheres to *C*. *albicans* yeast cell surface being kept under an enzymatically active form promoting the formation of EPS-rich biofilm matrix and enhancing accumulation of *S*. *mutans* on biofilms.^37^ Yet, *C.* a*lbicans*-bound GtfB produces more EPS than *S. mutans*-bound exoenzyme, leading to an enhanced EPS accumulation on biofilm matrix.^14^ On the other hand, downregulation of bacterial exoenzymes can occur in the presence of *C. albicans* leading to lack of EPS production by *S. mutans*^38^ which could also reduce the cariogenic potential of *S. mutans* biofilms.^22^ It is unclear though at this time the effect on three-dimensional structure of biofilms imposed by the present dual-species biofilm model. Further studies are necessary to assess the impact of the tested model and of the tested growth conditions on biofilm matrix production and its relation to carious lesion development.

## Conclusion

Overall, the data of the present study suggest that the presence of *C. albicans* can reduce the cariogenic potential of *S. mutans* biofilms when there is a competition for nutrients. Under this condition, *C. albicans* also modulates spent medium pH decreasing the acidogenic potential of *S. mutans* biofilms.
